# Neugebauer Models for Color Error Diffusion Halftoning

**DOI:** 10.3390/jimaging6040023

**Published:** 2020-04-17

**Authors:** Kaiming Wu, Kohei Inoue, Kenji Hara

**Affiliations:** Department of Communication Design Science, Kyushu University, Fukuoka 815-8540, Japan; wukaiming19941123@yahoo.co.jp (K.W.); hara@design.kyushu-u.ac.jp (K.H.)

**Keywords:** halftoning, error diffusion, sparse Neugebauer model

## Abstract

In this paper, we propose a method for halftoning color images based on an error diffusion technique, a color design criterion and Neugebauer models for expressing colors. For a natural extension of the conventional method for grayscale error diffusion to its color version, we first reformulate grayscale error diffusion with a one-dimensional Neugebauer model. Then we increase the dimension of the model to derive a color error diffusion method based on a three-dimensional Neugebauer model in RGB (red, green and blue) color space. Moreover, we propose a sparse Neugebauer model based on a color design criterion, or the minimal brightness variation criterion (MBVC), from which we derive a sparse Neugebauer model-based error diffusion method. Experimental results show that color halftone images produced by the proposed methods preserve the color contents in original continuous-tone images better than that by conventional color error diffusion methods. We also demonstrate that the proposed sparse method reduce halftone noise better than the state-of-the-art method based on MBVC.

## 1. Introduction

Digital halftoning is the process of converting a continuous-tone image into a pattern of a limited number of gray levels or colors. Recently, the techniques for digital halftoning have attracted the interest of researchers in the field of smart paper technology [1]. Smart paper is another name for the e-paper or electronic paper which is the electronic ink display technology designed to mimic the appearance of ordinary ink on paper. Electronic papers reflect light like ordinary papers, and can hold texts and images indefinitely without drawing electricity, while allowing the image to be changed later [2]. Therefore, it is said to be used for applications such as e-books, electronic newspapers, portable sings and foldable display [3]. Hsu et al. [4] mentioned the movie “Minority Report”, in which an electronic newspaper appears, and remarked that those technologies in the near future are becoming reality now. Kao et al. [5] proposed an integrated driving system for playing videos/animations on the electrophoretic display (EPD). The EPD system contains a real-time signal processing hardware engine for video halftoning based on Jarvis error diffusion. The proposed algorithm in this paper is a kind of error diffusion algorithms such as Jarvis error diffusion; therefore, the proposed algorithm can be applied to the EPD system by replacing the conventional error diffusion algorithm with the proposed one.

Error diffusion [6] is one of the typical digital halftoning techniques. Floyd and Steinberg [7] originated a grayscale error diffusion algorithm. A simple extension of grayscale error diffusion to color is to apply grayscale error diffusion to each color channel in a color image separately. These kinds of extensions are referred to as separable methods [8]. Shaked et al. formulated a color design criterion named the minimal brightness variation criterion (MBVC), and developed an ink relocation post-process applicable to arbitrary color halftone algorithms [9] and a color diffusion algorithm which decreases halftone noise successfully [10].

Neugebauer model for color representation can be used to infer the gamut boundaries of printed colorants on paper [11] by printers such as conventional CMYK (cyan, magenta, yellow and black) printers. Monga et al. [12] proposed an extension of tone-dependent error diffusion to color, which employs a Neugebauer printer model with a simple correction by Yule and Nielsen [13]. Inoue et al. [14] applied the Neugebauer model to the estimation of spectral reflectance.

In this paper, we propose a grayscale Neugebauer model for representing an arbitrary grayscale value by a linear combination of black and white, and apply it to grayscale error diffusion to derive a grayscale Neugebauer model-based method. Then we show the equivalence between conventional grayscale error diffusion and the Neugebauer model-based method. After that, we extend the grayscale Neugebauer model-based error diffusion method to its color version by extending the dimension of color space from 1D to 3D. Therefore, the proposed color error diffusion method can be expected as a good candidate for the color extensions of a standard grayscale error diffusion technique. Moreover, we propose a sparse Neugebauer model for improving the quality of halftone images. Experimental results show the effectiveness of the proposed color error diffusion method on real image data including standard images for image processing researches.

The rest of this paper is organized as follows: Section 2 summarizes grayscale error diffusion, and shows the equivalence between conventional grayscale error diffusion and Neugebauer model-based method. Section 3 extends grayscale Neugebauer model to its color version. Section 4 proposes a color Neugebauer model-based error diffusion method. Section 5 proposes a sparse color Neugebauer model-based error diffusion method. Section 6 shows experimental results for demonstrating the performance of the proposed methods. Finally, Section 7 concludes this paper.

## 2. Grayscale Error Diffusion

Let $F=[f_{ij}]$ be a grayscale image, where $f_{ij}\in \left[0,1\right]$ for i=1,2,…,m and j=1,2,…,n denotes the pixel value at the position (i,j) in *F*. Then conventional error diffusion method is described as follows.

### 2.1. Conventional Method

**Figure 1:**
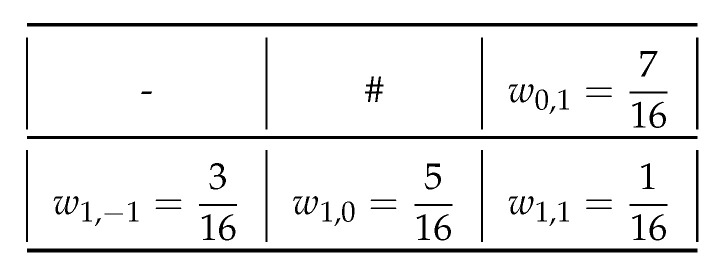
Error diffusion coefficients by Floyd–Steinberg [7].

Let $H^{F}=\left[h_{ij}^{F}\right]$ be a halftoned binary image of *F*. Then each pixel value $h_{ij}^{F}$ in $H^{F}$ is given by

(1) $$\begin{array}{c} h_{ij}^{F}=\left\{\begin{array}{cc} 1, & \mathrm{if}\;f_{ij}>\theta \\ 0, & \mathrm{otherwise} \end{array}\right. \end{array}$$

in raster scan order, where θ denotes a threshold value for binarization, and is given as $\theta =\frac{1}{2}$ in this paper. This binarization in Equation (1) causes an error as follows:

(2) $$\begin{array}{c} e_{ij}=f_{ij}-h_{ij}^{F} \end{array}$$

which is diffused to subsequent adjoining pixels as follows:

(3) $$\begin{array}{c} f_{i+k,j+l}^{\prime }=f_{i+k,j+l}+w_{kl}e_{ij} \end{array}$$

where such a pixel $f_{i+k,j+l}$ at the position (i+k,j+l) is updated to $f_{i+k,j+l}^{\prime }$ by adding the error $e_{ij}$ weighted by predetermined coefficients $w_{kl}$ as shown in Figure 1, where “#” denotes the current pixel being processed, and “-” denotes the past pixel in the raster scan order.

For subsequent pixels, the updated values $f_{ij}^{\prime }$ are binarized with Equation (1) instead of the original $f_{ij}$.

### 2.2. Grayscale Neugebauer Model-Based Method

**Figure 2:**
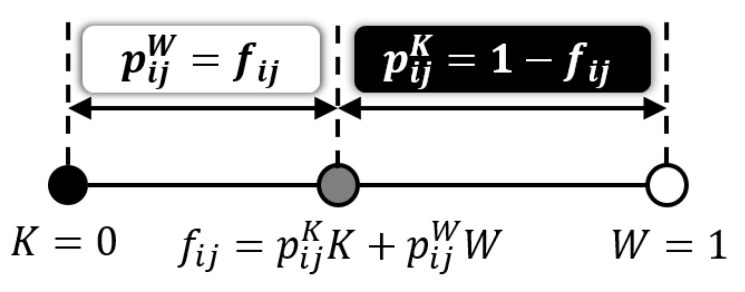
Grayscale Neugebauer model.

We present a grayscale Neugebauer model as follows:

(4) $$\begin{array}{c} f_{ij}=p_{ij}^{K}K+p_{ij}^{W}W \end{array}$$

where K=0 and W=1 denote black and white, respectively, and the coefficients defined by

(5) $$\begin{array}{cc} p_{ij}^{K} & =1-f_{ij} \end{array}$$

(6) $$\begin{array}{cc} p_{ij}^{W} & =f_{ij} \end{array}$$

can be viewed as the occurrence probabilities of black and white because of $p_{ij}^{K}\geq 0,\;p_{ij}^{W}\geq 0$ and $p_{ij}^{K}+p_{ij}^{W}=1$. In other words, the grayscale Neugebauer model expresses an arbitrary grayscale value $f_{ij}$ as an internally dividing point between black (*K*) and white (*W*) as illustrated in Figure 2.

Based on the grayscale Neugebauer model in Equation (4), we propose another expression of grayscale error diffusion as follows (the equivalence of these expressions will be discussed in the next subsection): A pixel value $h_{ij}^{F}$ in $H^{F}$ is given by selecting black or white which has larger probability than another as

(7) $$\begin{array}{c} h_{ij}^{F}=\arg\max_{x\in \{K,W\}}\left\{p_{ij}^{x}\right\} \end{array}$$

After this binarization, the occurrence probabilities become

(8) $$\begin{array}{cc} {\tilde{p}}_{ij}^{K} & =1-h_{ij}^{F} \end{array}$$

(9) $$\begin{array}{cc} {\tilde{p}}_{ij}^{W} & =h_{ij}^{F} \end{array}$$

using which, we define the errors in occurrence probabilities $p_{ij}^{K}$ and $p_{ij}^{W}$ as

(10) $$\begin{array}{cc} e_{ij}^{K} & =p_{ij}^{K}-{\tilde{p}}_{ij}^{K} \end{array}$$

(11) $$\begin{array}{cc} e_{ij}^{W} & =p_{ij}^{W}-{\tilde{p}}_{ij}^{W} \end{array}$$

which are diffused to the subsequent pixels as follows:

(12) $$\begin{array}{cc} {p\prime }_{i+k,j+l}^{K} & =p_{i+k,j+l}^{K}+w_{kl}e_{ij}^{K} \end{array}$$

(13) $$\begin{array}{cc} {p\prime }_{i+k,j+l}^{W} & =p_{i+k,j+l}^{W}+w_{kl}e_{ij}^{W} \end{array}$$

where the occurrence probabilities $p_{i+k,j+l}^{K}$ and $p_{i+k,j+l}^{W}$ of the subsequent adjoining pixel (i+k,j+l) are updated to ${p\prime }_{i+k,j+l}^{K}$ and ${p\prime }_{i+k,j+l}^{W}$, respectively.

For subsequent pixels, the updated values ${p\prime }_{ij}^{K}$ and ${p\prime }_{ij}^{W}$ are used for the binarization in Equation (7) instead of the original $p_{ij}^{K}$ and $p_{ij}^{W}$.

### 2.3. Equivalence of the Two Methods

Here we show an equivalence of the above two methods for grayscale error diffusion.

When $h_{ij}^{F}=K=0$ in Equation (7), we have $p_{ij}^{K}\geq p_{ij}^{W}$ or $1-f_{ij}\geq f_{ij}$, which means that $f_{ij}\leq \frac{1}{2}=\theta$, that is the condition for $h_{ij}^{F}=0$ in Equation (1). On the other hand, when $h_{ij}^{F}=W=1$ in Equation (7), we have $p_{ij}^{W}>p_{ij}^{K}$ or $f_{ij}>1-f_{ij}$, which means that $f_{ij}>\frac{1}{2}=\theta$, that is the condition for $h_{ij}^{F}=1$ in Equation (1). Therefore, the binarization in Equation (7) is equivalent to that in Equation (1).

Next, we describe the equivalence of error diffusion procedures. Assume that $h_{ij}^{F}=K=0$ in Equation (7). Then the occurrence probabilities in Equations (8) and (9) become ${\tilde{p}}_{ij}^{K}=1-h_{ij}^{F}=1$ and ${\tilde{p}}_{ij}^{W}=h_{ij}^{F}=0$, and the errors in Equations (10) and (11) become $e_{ij}^{K}=p_{ij}^{K}-{\tilde{p}}_{ij}^{K}=p_{ij}^{K}-1$ and $e_{ij}^{W}=p_{ij}^{W}-{\tilde{p}}_{ij}^{W}=p_{ij}^{W}$, respectively. Substituting these errors into Equations (12) and (13), we have

(14) $$\begin{array}{cc} {p\prime }_{i+k,j+l}^{K} & =p_{i+k,j+l}^{K}+w_{kl}\left(p_{ij}^{K}-1\right) \end{array}$$

(15) $$\begin{array}{cc} {p\prime }_{i+k,j+l}^{W} & =p_{i+k,j+l}^{W}+w_{kl}p_{ij}^{W} \end{array}$$

from both of which, using the relationships in Equations (5) and (6), we have

(16) $$\begin{array}{c} f_{i+k,j+l}^{\prime }=f_{i+k,j+l}+w_{kl}f_{ij} \end{array}$$

which coincides with Equation (3) with Equation (2) for $h_{ij}^{F}=K=0$.

On the other hand, when $h_{ij}^{F}=W=1$ in Equation (7), the occurrence probabilities in Equations (8) and (9) become ${\tilde{p}}_{ij}^{K}=1-h_{ij}^{F}=0$ and ${\tilde{p}}_{ij}^{W}=h_{ij}^{F}=1$, and the errors in Equations (10) and (11) become $e_{ij}^{K}=p_{ij}^{K}-{\tilde{p}}_{ij}^{K}=p_{ij}^{K}$ and $e_{ij}^{W}=p_{ij}^{W}-{\tilde{p}}_{ij}^{W}=p_{ij}^{W}-1$, respectively. Substituting these errors into Equations (12) and (13), we have

(17) $$\begin{array}{cc} {p\prime }_{i+k,j+l}^{K} & =p_{i+k,j+l}^{K}+w_{kl}p_{ij}^{K} \end{array}$$

(18) $$\begin{array}{cc} {p\prime }_{i+k,j+l}^{W} & =p_{i+k,j+l}^{W}+w_{kl}\left(p_{ij}^{W}-1\right) \end{array}$$

from both of which, using the relationships in Equations (5) and (6), we have

(19) $$\begin{array}{c} f_{i+k,j+l}^{\prime }=f_{i+k,j+l}+w_{kl}\left(f_{ij}-1\right) \end{array}$$

which also coincides with Equation (3) with Equation (2) for $h_{ij}^{F}=W=1$.

Consequently, the grayscale Neugebauer model-based error diffusion in Equations (12) and (13) can be reduced to the conventional error diffusion in Equation (3) with Equation (2), that shows the equivalence of the two methods.

## 3. Extending Grayscale Neugebauer Model to Higher Dimensions

**Figure 3:**
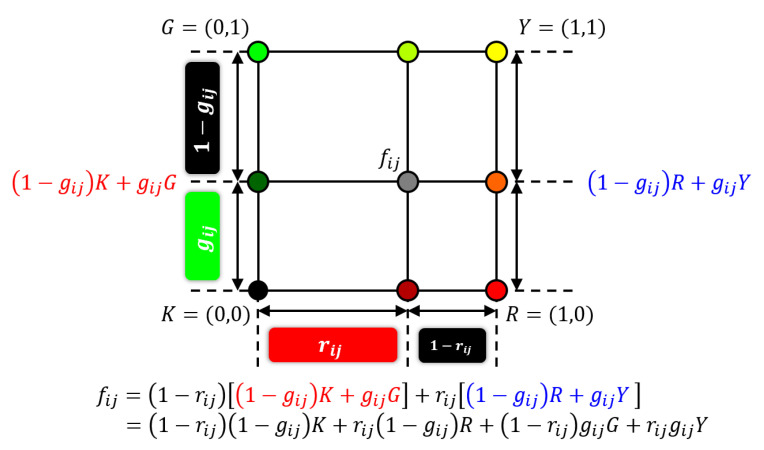
Two-dimensional Neugebauer model.

The grayscale Neugebauer model illustrated in Figure 2 has a one-dimensional structure, where a midpoint $f_{ij}$ is expressed as a linear interpolant between *K* and *W*. This one-dimensional structure of grayscale Neugebauer model can be extended to two-dimensional bilinear interpolation as follows: Let $f_{ij}=\left(r_{ij},g_{ij}\right)$ be a two-dimensional color vector on an RG (red and green) color plane as illustrated in Figure 3. Then $f_{ij}$ can be expressed as a bilinear interpolant of four basic colors: *K* (black), *R* (red), *G* (green) and *Y* (yellow) as shown in Figure 3, where a linear interpolant between *K* and *G* is expressed as $g_{ij}G+\left(1-g_{ij}\right)K$ for a given green value $g_{ij}$. Similarly, a linear interpolant between *R* and *Y* is expressed as $g_{ij}Y+\left(1-g_{ij}\right)R$. Using those expressions, $f_{ij}$ on the RG color plane can be expressed as a linear interpolant of $g_{ij}G+\left(1-g_{ij}\right)K$ and $g_{ij}Y+\left(1-g_{ij}\right)R$ for a given red value $r_{ij}$ as follows:

(20) $$\begin{array}{cc} f_{ij} & =\left(1-r_{ij}\right)\left[g_{ij}G+\left(1-g_{ij}\right)\;K\right]+r_{ij}\left[g_{ij}Y+\left(1-g_{ij}\right)\;R\right]\\ \; & =\left(1-r_{ij}\right)\left(1-g_{ij}\right)\;K+r_{ij}\left(1-g_{ij}\right)\;R+\left(1-r_{ij}\right)\;g_{ij}G+r_{ij}g_{ij}Y\\ \; & =p_{ij}^{K}K+p_{ij}^{R}R+p_{ij}^{G}G+p_{ij}^{Y}Y \end{array}$$

where four basic colors are given by K=(0,0),R=(1,0),G=(0,1) and Y=(1,1), and the coefficients are given by

(21) $$\begin{array}{cc} p_{ij}^{K} & =\left(1-r_{ij}\right)\left(1-g_{ij}\right) \end{array}$$

(22) $$\begin{array}{cc} p_{ij}^{R} & =r_{ij}\left(1-g_{ij}\right) \end{array}$$

(23) $$\begin{array}{cc} p_{ij}^{G} & =\left(1-r_{ij}\right)\;g_{ij} \end{array}$$

(24) $$\begin{array}{cc} p_{ij}^{Y} & =r_{ij}g_{ij} \end{array}$$

Thus we have a two-dimensional Neugebauer model in Equation (20) with Equations (21)–(24).

**Figure 4:**
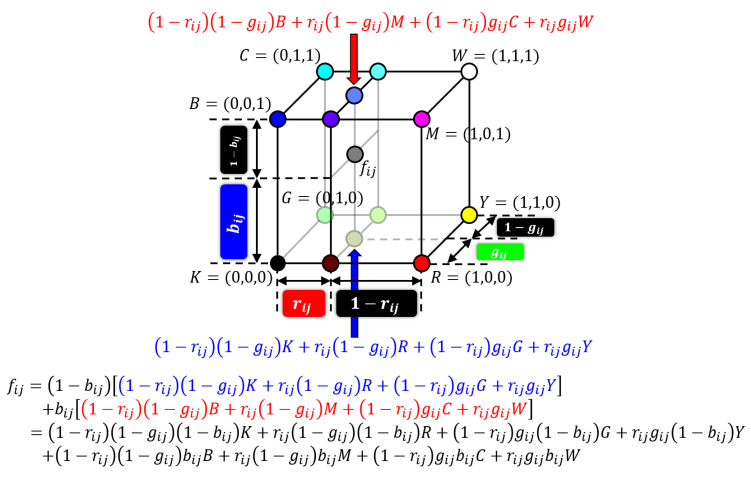
Three-dimensional Neugebauer model.

Next, we further extend the above two-dimensional Neugebauer model to three-dimensional one in order to deal with arbitrary colors in three-dimensional color space. Figure 4 illustrates the derivation of three-dimensional Neugebauer model as a trilinear interpolation of eight basic colors in RGB color space for representing an arbitrary color as a linear combination of the basic colors.

Let $f_{ij}=\left(r_{ij},g_{ij},b_{ij}\right)$ be a three-dimensional color vector in an RGB color space as shown in Figure 4. Then the orthogonal projection of $f_{ij}$ onto the bottom plane KRYG of the RGB color cube is given by Equation (20) using two-dimensional Neugebauer model. Similarly, another orthogonal projection of $f_{ij}$ onto the top plane BMWC of the RGB color cube is given by $\left(1-r_{ij}\right)\left(1-g_{ij}\right)B+r_{ij}\left(1-g_{ij}\right)M+\left(1-r_{ij}\right)g_{ij}C+r_{ij}g_{ij}W$. On the contrary, $f_{ij}$ can be represented as an interpolant of the above two orthogonal projections of $f_{ij}$ as follows:

(25) $$\begin{array}{cc} f_{ij}= & \left(1-b_{ij}\right)\left[\left(1-r_{ij}\right)\left(1-g_{ij}\right)\;K+r_{ij}\left(1-g_{ij}\right)\;R+\left(1-r_{ij}\right)\;g_{ij}G+r_{ij}g_{ij}Y\right]\\ \; & +b_{ij}\left[\left(1-r_{ij}\right)\left(1-g_{ij}\right)\;B+r_{ij}\left(1-g_{ij}\right)\;M+\left(1-r_{ij}\right)\;g_{ij}C+r_{ij}g_{ij}W\right]\\ = & \left(1-r_{ij}\right)\left(1-g_{ij}\right)\left(1-b_{ij}\right)\;K+r_{ij}\left(1-g_{ij}\right)\left(1-b_{ij}\right)\;R\\ \; & +\left(1-r_{ij}\right)\;g_{ij}\left(1-b_{ij}\right)\;G+r_{ij}g_{ij}\left(1-b_{ij}\right)\;Y\\ \; & +\left(1-r_{ij}\right)\left(1-g_{ij}\right)\;b_{ij}B+r_{ij}\left(1-g_{ij}\right)\;b_{ij}M\\ \; & +\left(1-r_{ij}\right)\;g_{ij}b_{ij}C+r_{ij}g_{ij}b_{ij}W\\ = & p_{ij}^{K}K+p_{ij}^{R}R+p_{ij}^{G}G+p_{ij}^{Y}Y+p_{ij}^{B}B+p_{ij}^{M}M+p_{ij}^{C}C+p_{ij}^{W}W \end{array}$$

where eight basic colors are given by K=(0,0,0),R=(1,0,0),G=(0,1,0),Y=(1,1,0),B=(0,0,1),M=(1,0,1),C=(0,1,1) and W=(1,1,1), and the coefficients are given by

(26) $$\begin{array}{cc} p_{ij}^{K} & =\left(1-r_{ij}\right)\left(1-g_{ij}\right)\left(1-b_{ij}\right) \end{array}$$

(27) $$\begin{array}{cc} p_{ij}^{R} & =r_{ij}\left(1-g_{ij}\right)\left(1-b_{ij}\right) \end{array}$$

(28) $$\begin{array}{cc} p_{ij}^{G} & =\left(1-r_{ij}\right)\;g_{ij}\left(1-b_{ij}\right) \end{array}$$

(29) $$\begin{array}{cc} p_{ij}^{Y} & =r_{ij}g_{ij}\left(1-b_{ij}\right) \end{array}$$

(30) $$\begin{array}{cc} p_{ij}^{B} & =\left(1-r_{ij}\right)\left(1-g_{ij}\right)\;b_{ij} \end{array}$$

(31) $$\begin{array}{cc} p_{ij}^{M} & =r_{ij}\left(1-g_{ij}\right)\;b_{ij} \end{array}$$

(32) $$\begin{array}{cc} p_{ij}^{C} & =\left(1-r_{ij}\right)\;g_{ij}b_{ij} \end{array}$$

(33) $$\begin{array}{cc} p_{ij}^{W} & =r_{ij}g_{ij}b_{ij} \end{array}$$

This three-dimensional Neugebauer model in Equation (25) with Equations (26)–(33) is used for color error diffusion in the next section.

## 4. Color Neugebauer Model-Based Method

Analogous to the grayscale Neugebauer model-based method in Section 2.2, we propose a color error diffusion method based on the color Neugebauer model in Section 3.

In color image halftoning, it is common to use a limited number of basic colors instead of true color. Let S={K,R,G,Y,B,M,C,W} be the set of eight basic colors. Then the color quantization of a pixel color $f_{ij}$ of an RGB color image F to one of the eight basic colors can be described as

(34) $$\begin{array}{c} h_{ij}^{F}=\arg\max_{x\in S}\left\{p_{ij}^{x}\right\} \end{array}$$

which is the corresponding pixel color in the color halftone image $H^{F}=\left[h_{ij}^{F}\right]$ of F, and $p_{ij}^{x}$ for x∈S are given by Equations (26)–(33). This expression in Equation (34) is a natural extension of grayscale binarization in Equation (7) to color quantization.

After this quantization, the occurrence probabilities become

(35) $$\begin{array}{c} {\tilde{p}}_{ij}^{x}=\left\{\begin{array}{cc} 1, & \mathrm{if}\;x=h_{ij}^{F}\\ 0, & \mathrm{otherwise} \end{array}\right. \end{array}$$

using which, we define the errors in occurrence probabilities $p_{ij}^{x}$ for x∈S as

(36) $$\begin{array}{c} e_{ij}^{x}=p_{ij}^{x}-{\tilde{p}}_{ij}^{x} \end{array}$$

which are diffused to the subsequent pixels as follows:

(37) $$\begin{array}{c} {p\prime }_{i+k,j+l}^{x}=p_{i+k,j+l}^{x}+w_{kl}e_{ij}^{x} \end{array}$$

where the occurrence probability $p_{i+k,j+l}^{x}$ of the subsequent adjoining pixel (i+k,j+l) is updated to ${p\prime }_{i+k,j+l}^{x}$.

For subsequent pixels, the updated values ${p\prime }_{ij}^{x}$ for x∈S are used for the quantization in Equation (34) instead of the original $p_{ij}^{x}$.

The above method is summarized in Algorithm 1.
**Algorithm 1** Color Neugebauer model-based error diffusion**Require:** RGB color image $F=[f_{ij}]$ where $f_{ij}=\left(r_{ij},g_{ij},b_{ij}\right)$**Ensure:** halftone image $H^{F}=\left[h_{ij}^{F}\right]$1:S←{K,R,G,Y,B,M,C,W}2:Ω←{(i,j)|i=1,2,…,mandj=1,2,…,n}3:**for**i←1 to *m* **do**4: **for** j←1 to *n* **do**5:  Compute coefficients $p_{ij}^{x}$ for x∈S by (26)–(33)6: **end for**7:**end for**8:**for**i←1 to *m* **do**9: **for** j←1 to *n* **do**10:  $h_{ij}^{F}\leftarrow \arg\max_{x\in S}\left\{p_{ij}^{x}\right\}$
11:  **for** x∈S **do**12:   ${\tilde{p}}_{ij}^{x}\leftarrow 1$ if $x=h_{ij}^{F}$ else 013:   $e_{ij}^{x}\leftarrow p_{ij}^{x}-{\tilde{p}}_{ij}^{x}$14:   **for** (k,l)∈{(0,1),(1,−1),(1,0),(1,1)} **do**15:    **if** (i+k,j+l)∈Ω **then**16:     $p_{i+k,j+l}^{x}\leftarrow p_{i+k,j+l}^{x}+w_{kl}e_{ij}^{x}$17:    **end if**18:   **end for**19:  **end for**20: **end for**21:**end for**22:**return**$H^{F}=\left[h_{ij}^{F}\right]$

In Algorithm 1, although we suppose that the error diffusion coefficients by Floyd–Steinberg [7] in Figure 1 is adopted, it is replaceable with other coefficients such as Jarvis–Judice–Ninke [15], Stucki [16] and Burkes [17].

## 5. Sparse Color Neugebauer Model-Based Method

The above Neugebauer model expresses an arbitrary color as a linear combination of eight basic colors in *S*. However, this expression is somewhat redundant, because a color in RGB color space exists in a tetrahedron, the vertices of which are selected from *S*, and therefore, four basic colors are sufficient for expressing an arbitrary color as a linear combination of basic colors.

**Figure 5:**
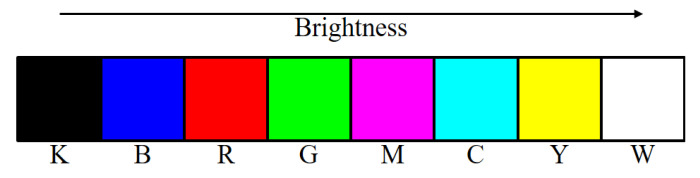
Eight basic colors ordered on the brightness axis.

Shaked et al. [10] proposed a high-quality color error diffusion method based on the MBVC, i.e., to reduce halftone noise, select from within all halftone sets by which the desired color may be rendered, the one whose brightness variation is minimal [9,10]. Based on MBVC, they derived the minimal brightness variation quadruples (MBVQs): {C,M,Y,W,},{M,Y,G,C},{R,G,M,Y},{K,R,G,B},{R,G,B,M} and {C,M,G,B}, which are the subsets of *S*, the elements of which are ordered on the brightness axis as shown in Figure 5. Although Shaked et al. [9] suggested a different brightness order, K,B,R,M,G,C,Y and *W*, for a CRT screen, we would like to use the order in Figure 5 for consistency with Shaked’s algorithm. That is, selecting a suitable brightness order is a problem-dependent on the display environment of colors.

**Figure 6:**
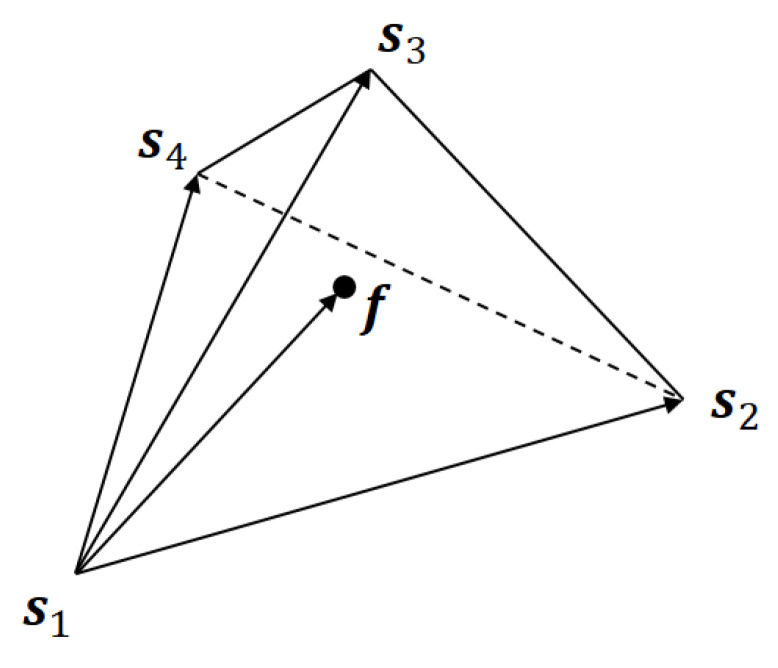
MBVQ $\left\{s_{1},s_{2},s_{3},s_{4}\right\}$ for a color f.

For a given RGB color, we can determine the corresponding MBVQ uniquely by Shaked’s algorithm. Let $S_{f}=\left\{s_{1},s_{2},s_{3},s_{4}\right\}\subset S$ be the MBVQ for a color f=(r,g,b). Then this situation can be illustrated as Figure 6, and we would like to express f as a linear combination of $S_{f}$ as:

(38) $$\begin{array}{c} f=p_{1}s_{1}+p_{2}s_{2}+p_{3}s_{3}+p_{4}s_{4} \end{array}$$

where $p_{1},\;p_{2},\;p_{3}$ and $p_{4}$ are nonnegative coefficients satisfying $p_{1}+p_{2}+p_{3}+p_{4}=1$.

As shown in Figure 6, the vector $f-s_{1}$ can be expressed as a linear combination of the vectors $s_{2}-s_{1},\;s_{3}-s_{1}$ and $s_{4}-s_{1}$ as follows:

(39) $$\begin{array}{c} f-s_{1}=p_{2}\left(s_{2}-s_{1}\right)+p_{3}\left(s_{3}-s_{1}\right)+p_{4}\left(s_{4}-s_{1}\right) \end{array}$$

Solving this simultaneous equations for $p_{2},\;p_{3}$ and $p_{4}$, we have the following expression of f:

(40) $$\begin{array}{c} f=\left(1-p_{2}-p_{3}-p_{4}\right)s_{1}+p_{2}s_{2}+p_{3}s_{3}+p_{4}s_{4} \end{array}$$

Putting $p_{1}=1-p_{2}-p_{3}-p_{4}$, we have a sparse Neugebauer model as follows:

(41) $$\begin{array}{c} f=\sum_{\xi =1}^{4}p_{\xi }s_{\xi }+\sum_{\eta =1}^{4}q_{\eta }t_{\eta } \end{array}$$

where $q_{\eta }=0$, and $t_{\eta }\in S\setminus S_{f}$ for η=1,2,3,4, i.e., every $t_{\eta }$ is the element of the complement of $S_{f}$ in *S*. By replacing the standard Neugebauer model in Algorithm 1 with the above sparse Neugebauer model, we propose another color error diffusion algorithm as Algorithm 2, where the function MBVQ(f) computes an MBVQ for a given color f by Shaked’s algorithm.
**Algorithm 2** Sparse Neugebauer model-based error diffusion**Require:** RGB color image $F=[f_{ij}]$ where $f_{ij}=\left(r_{ij},g_{ij},b_{ij}\right)$**Ensure:** halftone image $H^{F}=\left[h_{ij}^{F}\right]$1:S←{K,R,G,Y,B,M,C,W}2:Ω←{(i,j)|i=1,2,…,mandj=1,2,…,n}3:**for**i←1 to *m* **do**4: **for** j←1 to *n* **do**5:  Compute the MBVQ $S_{f}=\left\{s_{1},s_{2},s_{3},s_{4}\right\}\leftarrow \mathrm{MBVQ}\left(f\right)$ for each color $f\leftarrow (r_{ij},g_{ij},b_{ij})$6:  Solve the following simultaneous equations for $p_{2},\;p_{3}$ and $p_{4}$: $f-s_{1}=p_{2}\left(s_{2}-s_{1}\right)+p_{3}\left(s_{3}-s_{1}\right)+p_{4}\left(s_{4}-s_{1}\right)$7:  Compute $p_{1}\leftarrow 1-p_{2}-p_{3}-p_{4}$8:  **for** ξ←1 to 4 **do**9:   $p_{ij}^{s_{\xi }}\leftarrow p_{\xi }$10:  **end for**11:  Compute $\left\{t_{1},t_{2},t_{3},t_{4}\right\}\leftarrow S\setminus S_{f}$12:  **for** η←1 to 4 **do**13:   $p_{ij}^{t_{\eta }}\leftarrow 0$14:  **end for**15: **end for**16:**end for**17:**for**i←1 to *m* **do**18: **for** j←1 to *n* **do**19:  $h_{ij}^{F}\leftarrow \arg\max_{x\in S}\left\{p_{ij}^{x}\right\}$20:  **for** x∈S **do**21:   ${\tilde{p}}_{ij}^{x}\leftarrow 1$ if $x=h_{ij}^{F}$ else 022:   $e_{ij}^{x}\leftarrow p_{ij}^{x}-{\tilde{p}}_{ij}^{x}$23:   **for** (k,l)∈{(0,1),(1,−1),(1,0),(1,1)} **do**24:    **if** (i+k,j+l)∈Ω **then**25:     $p_{i+k,j+l}^{x}\leftarrow p_{i+k,j+l}^{x}+w_{kl}e_{ij}^{x}$26:    **end if**27:   **end for**28:  **end for**29: **end for**30:**end for**31:**return**$H^{F}=\left[h_{ij}^{F}\right]$

## 6. Experimental Results

**Figure 7:**
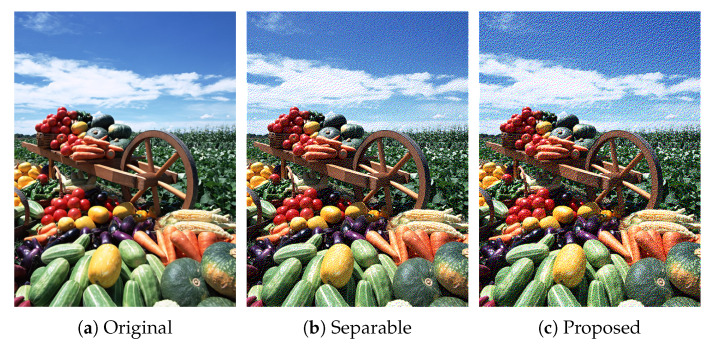
Color image halftoning.

In this section, we demonstrate the performance of the proposed error diffusion methods using real images. Figure 7 shows an example of color image halftoning, where an original color image in Figure 7a is halftoned by conventional separable error diffusion method as shown in Figure 7b. The separable method [8] applies grayscale error diffusion to each color channel separately, and combines the resultant three grayscale halftone images into a single color halftone image. Figure 7c shows the result of the proposed color Neugebauer model-based error diffusion method summarized in Algorithm 1. Although Figure 7b,c look similar to each other from a distance, a close look at these images reveals that Algorithm 1 loses the clarity of the reproduction of contours. Algorithm 2 will improve the quality of contour reproduction as shown in the following results.

**Figure 8:**
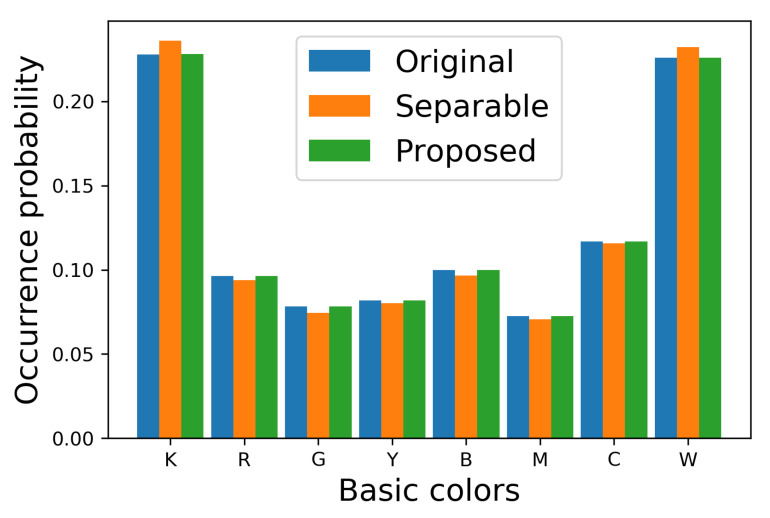
Occurrence probabilities of basic colors.

In order to show the difference between Figure 7b,c clearly, we show the occurrence probabilities of eight basic colors (K=(0,0,0),R=(1,0,0),G=(0,1,0),Y=(1,1,0),B=(0,0,1),M=(1,0,1),C=(0,1,1) and W=(1,1,1)) in each image in Figure 8, where the vertical and horizontal axes denote the occurrence probability and the basic colors, respectively, where the occurrence probability of a basic color x∈S is defined by

(42) $$\begin{array}{c} p^{x}=\frac{1}{mn}\sum_{i=1}^{m}\sum_{j=1}^{n}p_{ij}^{x} \end{array}$$

**Figure 9:**
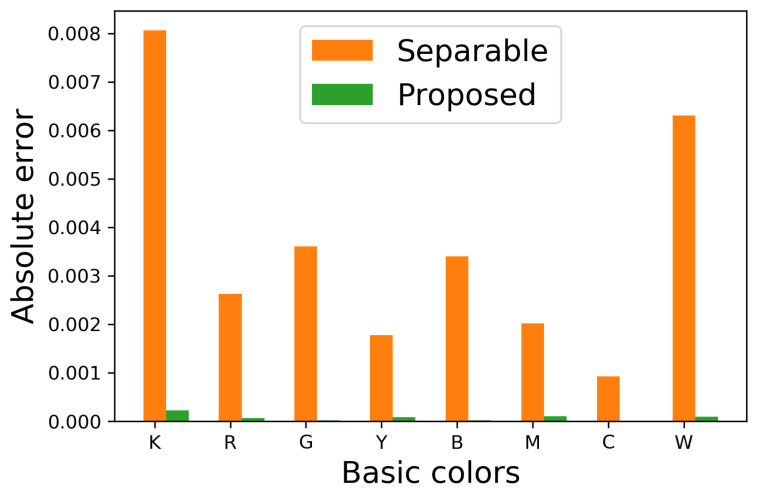
Absolute errors in occurrence probabilities in Figure 8.

Blue, orange and green bars denote the original image and the halftoned images by the separable and the proposed methods, respectively. This figure shows that the occurrence probabilities for the separable method deviate from the original ones, on the other hand, that for the proposed method follow the original ones well. To confirm this difference quantitatively, we compute the absolute error of the occurrence probabilities defined by

(43) $$\begin{array}{c} E\left(p_{\mathrm{Orig}}^{x},p_{\mathrm{Half}}^{x}\right)=\left|p_{\mathrm{Orig}}^{x}-p_{\mathrm{Half}}^{x}\right| \end{array}$$

for x∈S, where $p_{\mathrm{Orig}}^{x}$ and $p_{\mathrm{Half}}^{x}$ denote the occurrence probabilities of basic color x in an original and its halftoned images, respectively. Figure 9 shows $E(p_{\mathrm{Orig}}^{x},p_{\mathrm{Half}}^{x})$ for eight basic colors, where orange and green bars denote that the occurrence probabilities $p_{\mathrm{Half}}^{x}$ are computed from the halftoned images by the separable and proposed methods, respectively. This figure shows that the proposed method can decrease the absolute error compared with the separable method.

**Figure 10:**
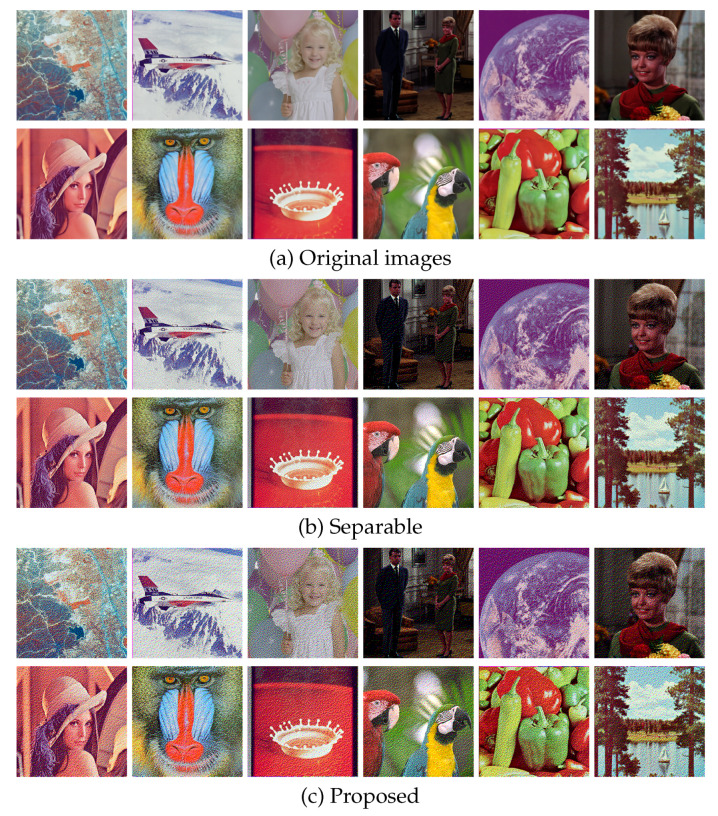
Results on the SIDBA images.

**Figure 11:**
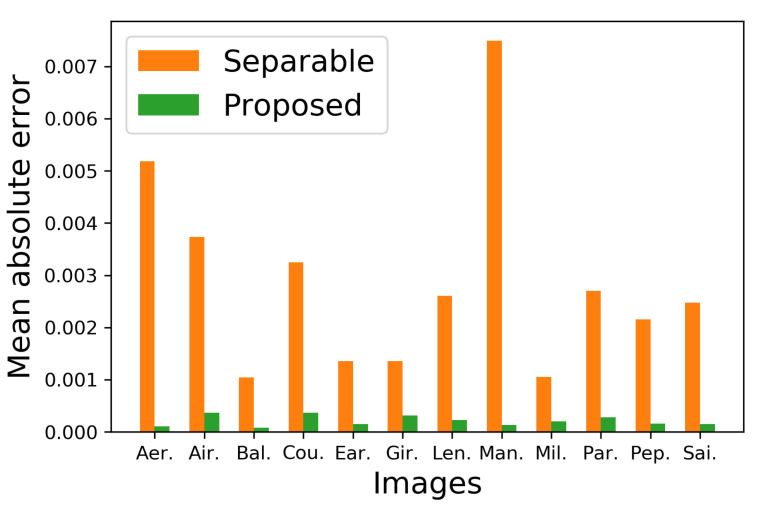
Mean absolute errors of halftoned SIDBA images in Figure 10.

Figure 10 shows additional results on the standard image database SIDBA [18], which contains twelve color images as shown in Figure 10a. Figure 10b,c show the results of the separable and the proposed methods, respectively. Figure 11 shows the mean absolute error of the occurrence probabilities of the eight basic colors, where the vertical and horizontal axes denote the mean absolute error and the twelve images shown in Figure 10, respectively. The proposed method denoted by green bars achieved smaller errors compared with the separable method denoted by orange bars for all twelve SIDBA images.

**Figure 12:**
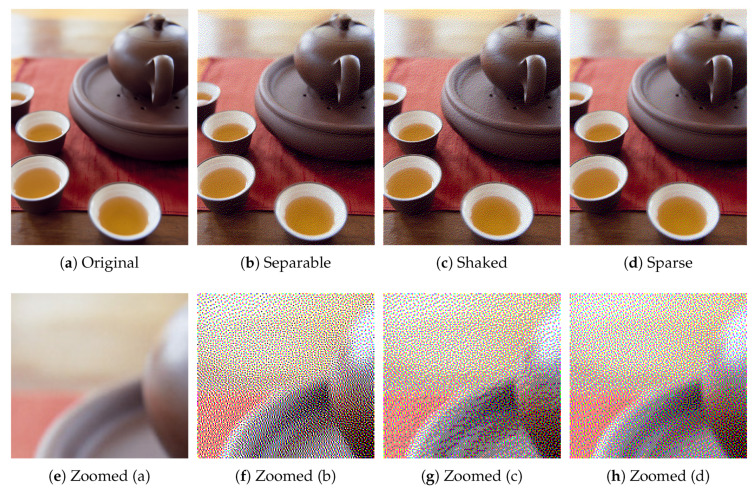
Color image halftoning with zoomed parts.

Next, we compare the proposed sparse Neugebauer model-based method (Algorithm 2) with Shaked’s method [10]. Figure 12 compares conventional separable, Shaked’s and the proposed sparse methods, where Figure 12a shows an original color image, which is halftoned by the separable, Shaked’s and proposed methods as Figure 12b–d, respectively. Their zoomed parts are also shown at the bottom of them.

As shown in Figure 12f, separable method places dark and light pixels side by side with each other here and there, which conflicts with the MBVC. On the other hand, Shaked’s method reduces the brightness variation, however, the subtle gradations on the surface of ceramic ware are disturbed in Figure 12g. Compared with Shaked’s method, the proposed method achieves both the brightness variation reduction and the subtle gradation reproduction as shown in Figure 12h.

**Figure 13:**
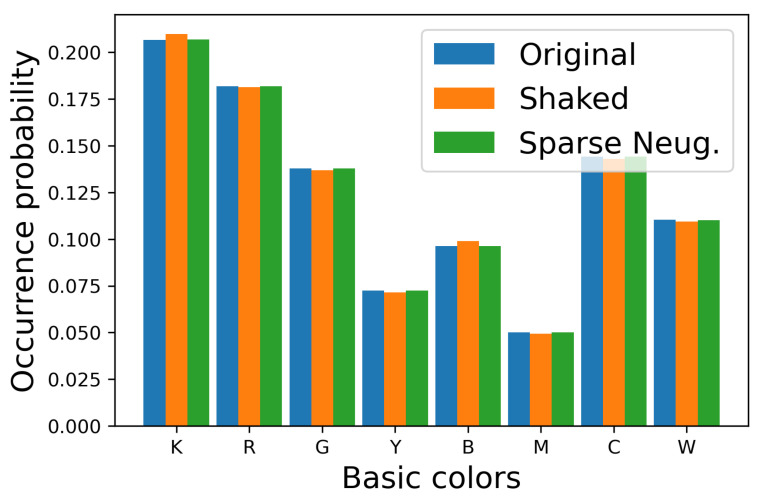
Occurrence probabilities of basic colors.

For evaluating the quality of halftoned images in Figure 12 quantitatively, we show the occurrence probabilities of eight basic colors in each image in Figure 13, where the vertical and horizontal axes denote the occurrence probability and the basic colors, respectively. Blue, orange and green bars denote the original image and the halftoned images by Shaked’s and the proposed methods, respectively.

**Figure 14:**
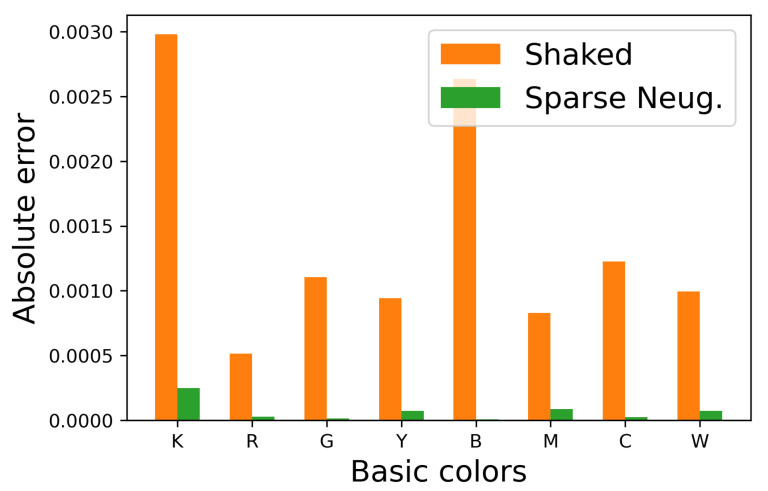
Absolute errors in occurrence probabilities in Figure 13.

This figure shows that the occurrence probabilities for the Shaked method deviate from the original ones, on the other hand, that for the proposed sparse method follows the original ones well. To confirm this difference quantitatively, we show the absolute error of the occurrence probabilities in Figure 14, which shows that the proposed sparse method can decrease the absolute error compared with Shaked’s method.

**Figure 15:**
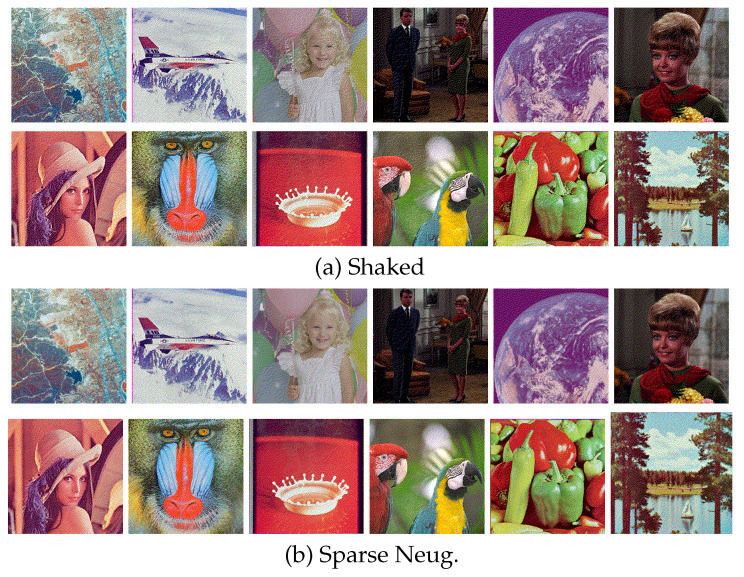
Halftoned SIDBA images.

Figure 15 shows halftoned SIDBA images by Shaked’s and the proposed sparse methods, which produce visually similar halftone images to each other.

**Figure 16:**
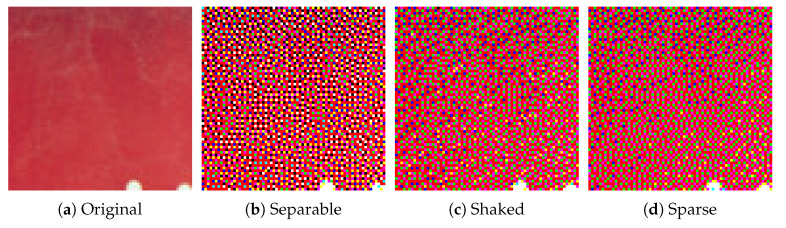
Zoomed parts of Milkdrop images.

However, the produced halftone images are not identical as shown in Figure 16, where Figure 16a–d show the zoomed parts of the same subregions of Milkdrop images in Figure 10a,b, and Figure 15a,b, respectively. The original patch in Figure 16a is halftoned by the separable method as shown in Figure 16b, where all eight basic colors are used to reproduce the original colors. In Figure 16c, Shaked’s method succeeds in reducing black and white pixels for minimizing the brightness variation. However, in Figure 16c, the second darkest blue and the second lightest yellow pixels are still intermingled spatially. On the other hand, in Figure 16d, the proposed sparse method reduces the spatial mixture of blue and yellow pixels compared with Figure 16c. This result demonstrates that the proposed sparse method (Algorithm 2) can separate different colors spatially on the image plane better than Shaked’s method, which improves the clarity of the reproduction of contours, and therefore, Algorithm 2 can also be applicable to the reproduction of textual information as well as Shaked’s method.

**Figure 17:**
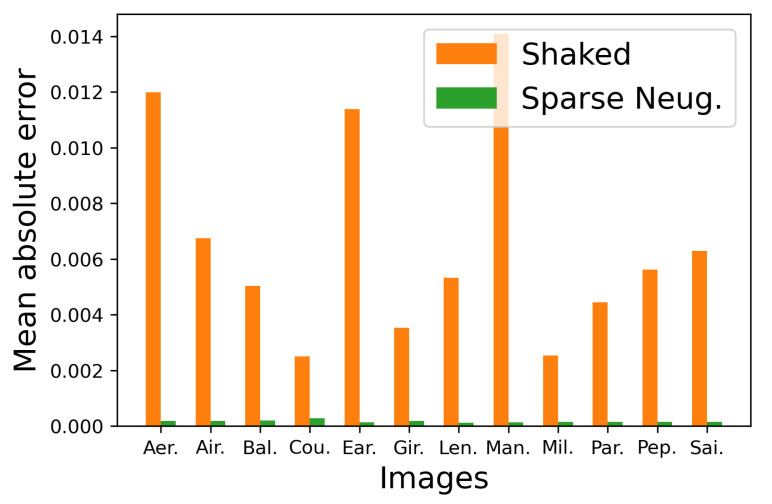
Mean absolute errors of halftoned SIDBA images in Figure 15.

To confirm the difference between Shaked’s and the proposed sparse methods quantitatively, we show the mean absolute error of the occurrence probabilities of the eight basic colors in Figure 17, where the proposed sparse method denoted by green bars achieved smaller errors compared with the Shaked’s method denoted by orange bars for all twelve SIDBA images.

## 7. Conclusions

In this paper, we proposed a method for color error diffusion by extending the grayscale Neugebauer model to its color version. The color Neugebauer model represents a color by a linear combination of basic colors, where the coefficients can be interpreted as the occurrence probabilities of the basic colors because they satisfy the probability axioms.

We also proposed a sparse Neugebauer model for color error diffusion halftoning. The proposed sparse method is based on the MBVC proposed by Shaked et al. Therefore, the proposed sparse method can also reduce halftone noise as well as Shaked’s method.

Experimental results demonstrated that the color halftone images given by the proposed method preserve the occurrence probabilities of the basic colors of the original images well. Compared with Shaked’s method, the proposed sparse method achieved a better performance both visually and quantitatively.

## Abbreviations

The following abbreviations are used in this manuscript:

MBVC	Minimal Brightness Variation Criterion
MBVQ	Minimal Brightness Variation Quadruples
